# Blood Urea Nitrogen as a Predictor of Severe Acute Pancreatitis Based on the Revised Atlanta Criteria: Timing of Measurement and Cutoff Points

**DOI:** 10.1155/2017/9592831

**Published:** 2017-04-13

**Authors:** Suhan Lin, Wandong Hong, Zarrin Basharat, Qipin Wang, Jingye Pan, Mengtao Zhou

**Affiliations:** ^1^Department of Gastroenterology and Hepatology, The First Affiliated Hospital of Wenzhou Medical University, Wenzhou, Zhejiang, China; ^2^School of the First Clinical Medical Sciences, Wenzhou Medical University, Wenzhou, Zhejiang, China; ^3^Microbiology & Biotechnology Research Lab, Department of Environmental Sciences, Fatima Jinnah Women University, Rawalpindi 46000, Pakistan; ^4^Intensive Care Unit, The First Affiliated Hospital of Wenzhou Medical University, Wenzhou, Zhejiang, China; ^5^Department of Surgery, The First Affiliated Hospital of Wenzhou Medical University, Wenzhou, Zhejiang, China

## Abstract

*Background and Aims*. This study evaluated the prognostic accuracy of BUN for severe acute pancreatitis (SAP) and in-hospital mortality (IHM) in terms of the best timing for BUN measurement and the optimal BUN cutoff points.* Methods*. BUN determinants at the time of admission and 24 hrs after hospital admission were recorded and analyzed statistically. The ability of BUN in predicting the SAP and the occurrence of IHM were assessed using the area under the receiver-operating characteristic (ROC) curve.* Results*. For SAP, AUC of BUN at admission and 24 hrs after hospital admission was 0.75 and 0.80, respectively. For IHM in acute pancreatitis, it was 0.86 at admission and 0.84 after 24 hrs of hospital admission, respectively. The optimal cutoff point of BUN 24 hrs after hospital admission for SAP and at admission for IHM was 8.3 mmol/L and 13.3 mmol/L, respectively.* Conclusion.* BUN determination after 24 hrs of hospital admission has high accuracy for prediction of SAP while BUN at initial admission has high accuracy for prediction of IHM.

## 1. Introduction

Acute pancreatitis (referred to hereafter as AP) is a common clinical condition with variation in severity [1]. In most instances, AP runs a self-limiting and mild course; however, 10–30% of patients might progress to a severe attack, that is, severe acute pancreatitis (SAP) with an in-hospital mortality (IHM) reaching 30% [2, 3]. SAP, representing a paradigm of sterile inflammatory disease, is initiated by acinar cell injury-mediated local inflammation and progresses to the systemic inflammatory response syndrome, accompanied with inevitable multiple organ injury, and eventually leads to multiple organ dysfunction syndrome, which contributes to the main cause of morbidity and mortality in this condition [4]. Moreover, this typical pathological course results in approximately 50% of clinical deaths within the first week [5].

The early risk assessment of patients with AP through reliable methods is necessary to improve the clinical outcomes and reduce the treatment cost and length of hospitalization. A great deal of research has focused on development of approaches for early diagnosis and risk stratification in AP. In addition to the established clinical scores, a variety of single markers have emerged for AP, their measurement being sufficiently fast and cheap to widely enter the clinical routine. The multiparametric scores were essential for clinical trials but had obvious disadvantages that prevent their practical use in daily routine [6].

Blood urea nitrogen (BUN) is an important prognostic marker during the initial 24 hours of hospitalization for measuring SAP and IHM [7–10]. In the literature, there is no consensus on the timing of measurement of BUN for prediction of SAP and IHM during the first 24 hrs of hospitalization [7, 8]. There is also inconsistency in the optimal cutoff points for BUN in terms of predicting IHM of AP [6–8, 11]. In addition, BUN in previous studies was evaluated as a predictor of severe pancreatitis, defined according to the Atlanta criteria but not based on the recent SAP guidelines [12], which limit its use in the current clinical practice. Therefore, the current study aimed to assess the best timings for measurement and the optimal cutoff points of BUN in predicting SAP. This was defined by up-to-date guidelines and IHM in AP.

## 2. Material and Methods

### 2.1. Patient Population, Data Collection, and Ethics

Patients aged 18 years or older, admitted with a diagnosis of AP (onset time ≤ 3 days) in the First Affiliated Hospital of Wenzhou Medical University between January 2013 and December 2015, were eligible for study participation. Patients were excluded from participation in case they met any of the following criteria: onset time >3 days (515 cases), not-first-time pancreatitis (194 cases), therapeutic operations (23 cases), organ failure before data collection (including history of cirrhosis/chronic kidney disease with creatinine clearance <40 mL/min/pulmonary disease) (7 cases), malignant gastrointestinal tumors (19 cases), gestation (7 cases), intoxication (5 cases), and merging the above conditions (27 cases).

The following information was collected for each patient: age, gender, Body Mass Index (BMI), etiology, white blood cell (WBC), hemoglobin (Hb), platelet count (PLT), and serum electrolytes (potassium, sodium, and calcium) at admission. BUN and serum creatinine (Scr) were collected at the time of admission and 24 hrs after hospital admission.

This study protocol was approved by the Ethic Committee of the First Affiliated Hospital of Wenzhou Medical University. This study was performed according to the principles expressed in the Declaration of Helsinki and informed consent was obtained from the subjects.

### 2.2. Definitions and Outcomes

The diagnosis of AP requires two of the following three features in the revised Atlanta criteria [12]: (1) abdominal pain; (2) level of serum lipase or amylase at least three times greater than the upper limit of normal; and (3) characteristic findings of AP on abdominal image. According to the revised Atlanta criteria, SAP is defined as persistent organ failure in patients (organ failure persistent for more than 48 h). According to the modified Marshall Scoring System [13], organ failure includes at least one of the following features: (a) respiratory failure, defined as PaO_2_/FiO_2_ levels of 300 mmHg or less; (b) renal failure, defined as Scr level of at least 1.9 mg/dl; and (c) shock, defined as systolic blood pressure of less than 90 mmHg and unresponsive to fluid therapy. IHM refers to death occurring from AP or its complications during the initial hospitalization.

The primary endpoint was to assess BUN as predictor of SAP at initial admission and 24 hrs after patient admission. The secondary endpoint was to assess BUN as predictor of IHM at initial admission and 24 hrs after patient admission in the hospital.

### 2.3. Statistical Analysis

A Shapiro–Wilk test was used to evaluate whether the continuous data was normally distributed. According to the results of the Shapiro–Wilk test, continuous values were expressed by mean ± SD or median and interquartile range. Values were compared using the paired* t*-test or the Wilcoxon signed-rank test. Categorical values were described by count and proportions and compared by the *χ*^2^ test.* P* values below 0.05 were considered significant.

Variables in univariate analysis found to be significantly related to SAP were selected as candidates for ROC curve analysis. AUC for BUN as a predictor of SAP and IHM was calculated. A predictor with an AUC above 0.7 was considered useful, while an AUC between 0.8 and 0.9 indicated excellent diagnostic accuracy [14]. To determine the optimal BUN cutoff points for SAP and IHM, Youden Index, sensitivity, specificity, positive predictive value (PPV), and negative predictive value (NPV) were calculated for the different BUN cutoff points. The best Youden Index (sensitivity + specificity − 1) was used to determine the best cutoff points of BUN level to predict SAP and IHM [15, 16].

*P* values below 0.05 were considered significant. All statistical procedures were performed with Stata 12.0. software.

## 3. Results

### 3.1. Patient Characteristics

A total of 671 participants were included in the current study. The median age of the patients was 47 years (range 19–93 years), and 62.44%  (*n* = 419) of patients were males. Biliary disease was the most common cause of the AP (*n* = 284, 42.32%). 60 (8.94%) patients presented with severe AP symptoms. The median length of the hospital stay was 10 days (interquartile range 7–14 days), with 13 days (interquartile range 7–24 days) for SAP patients. Eleven patients (1.64%) died during hospitalization.

### 3.2. Univariate Analysis between Patients with or without SAP

Fourteen variables were considered potentially relevant to the severity of AP and tested using univariable analysis. As shown in Table 1, hemoglobin at admission, platelets at admission, WBC at admission, calcium, BUN at admission, BUN 24 hrs after hospital admission, Scr at admission, and Scr after 24 hrs of hospital admission were significantly associated with the severity of AP.

### 3.3. Prognostic Values of BUN in Predicting SAP

**Figure 1:**
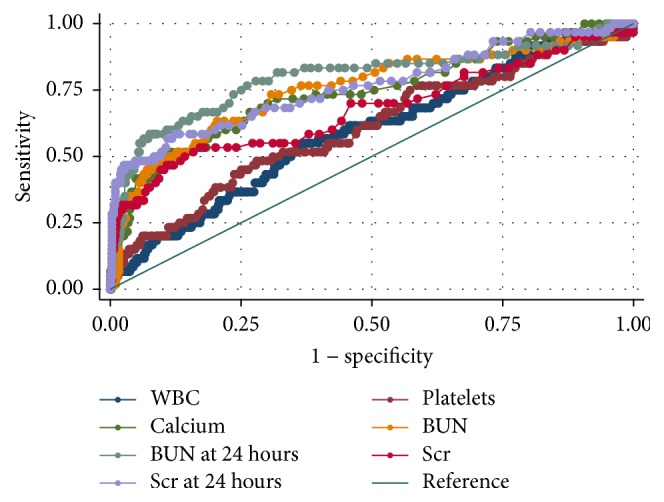
ROC curves for various predictors for SAP. The AUCs were 0.59 ± 0.08, 0.61 ± 0.08, 0.74 ± 0.08, 0.75 ± 0.08, 0.80 ± 0.07, 0.68 ± 0.09, and 0.76 ± 0.08 for WBC at admission, platelets at admission, calcium at admission, BUN at admission, BUN after 24 hrs of hospitalization, Scr at admission, and Scr after 24 hrs of hospitalization, respectively. The ideal AUC was 1.00. The reference line represents AUC of 0.50, based on chance alone.

As shown in Figure 1, the AUCs for WBC at admission, platelets at admission, calcium at admission, BUN at admission, BUN after 24 hrs of admission, Scr at admission, and Scr after 24 hrs of admission for the prediction of SAP were 0.59 ± 0.08, 0.61 ± 0.08, 0.74 ± 0.08, 0.75 ± 0.08, 0.80 ± 0.07, 0.68 ± 0.09, and 0.76 ± 0.08, respectively. Both the BUN at admission and BUN after 24 hours of hospitalization were useful predictors of SAP, with AUC of more than 0.7. In addition, only BUN after 24 hours of hospitalization with an AUC of 0.80 achieved an excellent diagnostic accuracy among single predictors.

Every cutoff point of BUN at admission and after 24 hrs for prediction of SAP was shown in Tables 2 and 3. Based on Youden Index analysis, the optimal BUN cutoff point at admission and after 24 hrs of hospital admission for prediction of SAP was 6.1 mmol/L (17.1 mg/dL) and 8.3 mmol/L (23.2 mg/dL), respectively.

### 3.4. Prognostic Values of BUN in Predicting IHM of AP

**Figure 2:**
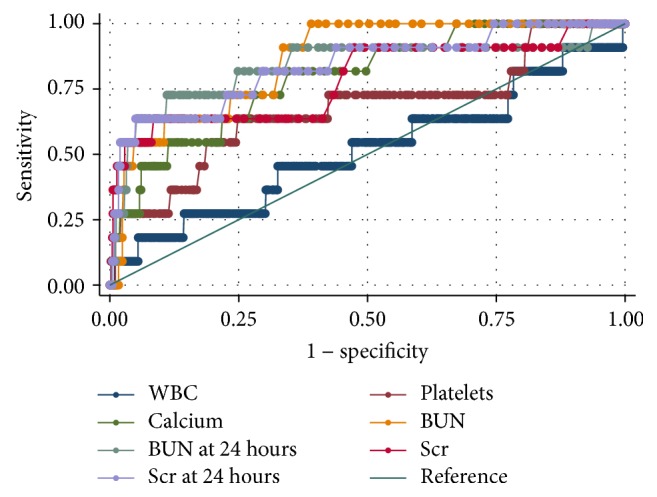
ROC curves for various predictors for IHM. The areas under ROC curves were 0.52 ± 0.20, 0.68 ± 0.19, 0.79 ± 0.13, 0.86 ± 0.09, 0.84 ± 0.16, 0.79 ± 0.175, and 0.84 ± 0.14 for WBC at admission, platelets at admission, calcium at admission, BUN at admission, BUN after 24 hrs of hospitalization, Scr at admission, and Scr after 24 hrs of hospitalization, respectively. The ideal AUC was 1.00. The reference line represents AUC of 0.50, based on chance alone.

As shown in Figure 2, the AUCs for WBC at admission, platelets at admission, calcium at admission, BUN at admission, BUN after 24 hrs of admission, Scr at admission, and Scr after 24 hrs of admission, for the prediction of SAP, were 0.52 ± 0.20, 0.68 ± 0.19, 0.79 ± 0.13, 0.86 ± 0.09, 0.84 ± 0.16, 0.79 ± 0.175, and 0.84  ±  0.14, respectively. Both the BUN at admission and BUN after 24 hours of hospitalization were excellent predictors of IHM due to AP, with AUC of more than 0.8. In addition, BUN at admission achieved the biggest AUC (0.86) among single predictors of IHM of AP.

Every cutoff point of BUN for prediction of IHM due to AP is shown in Tables 4 and 5. With a cutoff point of 5.2 mmol/L (14.6 mg/dL) at admission and 8.3 mmol/L (23.2 mg/dL) at 24 hrs after hospital admission, BUN achieved the best Youden Index for prediction of IHM. However, these cutoff points result in a very low PPV (only 4% at admission and 9.9% at 24 hrs after hospital admission) for IHM. One may expect to choose another more pragmatic cutoff with a higher PPV for the trade-off of slightly lower NPV (which would still be about 90%) for BUN to predict IHM. Therefore, 13.3 mmol/L (37.3 mg/dL) and 12 mmol/L (34 mg/dL) were selected as the optimal BUN cutoff point at admission and 24 hrs after hospital admission for prediction of IHM in AP.

## 4. Discussion

The outcomes of the present study prove that (i) both the BUN at admission and that after 24 hrs of hospitalization were useful predictors of SAP, with AUC of more than 0.7. In addition, only BUN after 24 hours of hospitalization with an AUC of 0.80 achieved an excellent diagnostic accuracy among single predictors; (ii) the optimal BUN cutoff point at admission and 24 hrs after hospital admission for prediction of SAP was 6.1 mmol/L (17.1 mg/dL) and 8.3 mmol/L (23.2 mg/dL), respectively; (iii) both BUN at admission and that after 24 hrs of hospitalization were excellent predictors of IHM of AP, with AUC of more than 0.8. In addition, BUN at admission achieved the largest AUC (0.86) among single predictors of IHM of AP; (iv) the optimal cutoff point of BUN at admission and at 24 hrs after hospital admission for prediction of IHM of AP was 13.3 mmol/L (37.3 mg/dL) and 12 mmol/L (34 mg/dL).

There are a lot of grounds, based on which BUN has been selected as useful prognostic/predictor of AP in the literature. First, an ideal prognostic marker should help guide physicians in their approach to accepted interventions such as fluid resuscitation. The BUN at admission/hospitalization might reflect the underlying physiologic state of the patient, including intravascular volume depletion [11] and prerenal azotemia [7]. Therefore, BUN may play an important role in the early assessment of AP [7]. Second, a persistent elevation or subsequent rise in BUN may reflect either a failure to adequately resuscitate patients early in their disease course, deterioration of renal function, or a state of ongoing negative nitrogen balance related to increased protein catabolism induced by AP [17]. Third, it has been suggested that, though without clinical signs of pancreatitis and changes of medical image, the fluctuation of BUN is particularly susceptible to the ischemic injury of pancreas [18]. Besides the influence of pancreatic enzymes, inflammatory factors (such as IL-1*β* and IL-18) could cause renal dysfunction through cardiac dysfunction, circulatory collapse, hypoperfusion, metabolic acidosis, shock and production of acute respiratory distress syndrome indirectly, and direttissima after SAP induction [19, 20].

Previous study reported that elevated BUN level (more than 25 mg/dl) at admission was associated with increased possibility of developing severe AP defined by Atlanta criteria [21]. It achieved a sensitivity of 27.3%, specificity of 97.7%, positive predictive value of 78.3%, and negative predictive value of 81.3% [21]. To the best of our knowledge, this is the first study to evaluate BUN as predictor of SAP defined by the up-to-date revised Atlanta criteria. Our univariate analysis suggested that there were statistical differences in BUN level both at admission and after 24 hours of admission between patients with or without SAP (Table 1). Every cutoff point of BUN at admission and after 24 hrs for prediction of SAP was evaluated (Tables 2 and 3). Our study noted that BUN after 24 hours of hospitalization had higher AUC than that of BUN at initial admission (Figure 1). With a cutoff of 8.3 mmol/L (23.2 mg/dL), BUN at 24 hours of hospitalization achieved a sensitivity of 0.508, specificity of 0.925, PPV of 0.432, and NPV of 0.958.

The early phase of mortality peak of this dynamic disease usually lasts for the first week, with approximately 50% clinical death occurrence during the first week [5]. Previous studies suggested that elevated BUN and subsequent changes of BUN during the initial 24 hrs of hospitalization are the most valuable independent risk factors for mortality in AP [7–10]. Different studies have proposed different optimal cutoff points of BUN at admission, ranging from 33 mg/dl [6], 25 mg/dl [22], 23 mg/dl [10], and 20 mg/dl [8] to 7.8 mg/dl [7], for prediction of IHM. In one study, the cutoff point of BUN, that is, 7.8 mg/dl, at admission was associated with a corresponding increase in risk of mortality (OR = 2.9) [7]. BUN level in patients at initial admission that declined by 5 mg/dl or more after 24 hrs had a substantially reduced mortality risk (0%–3.2%) [8]. Any rise in BUN level at 24 hours was associated with an OR of 4.3 (CI_95_, 2.3–7.9%) for death [8]. Our data suggested that both the BUN at admission and BUN at 24 hrs of hospitalization were excellent predictors of IHM of AP. In addition, BUN at admission achieved the largest AUC (i.e., 0.86) among single predictors of IHM of AP (Figure 2). The optimal cutoff point of BUN at admission and at 24 h after hospital admission for prediction of IHM of AP was 13.3 mmol/L (37.3 mg/dL) and 12 mmol/L (34 mg/dL), respectively (Tables 4 and 5). With a cutoff value of 13.3 mmol/L (37.3 mg/dL), BUN at admission achieved a sensitivity of 0.455, specificity of 0.977, PPV of 0.217, and NPV of 0.991. Low mortality might be one of primary reasons why large fluctuations of the optimal cutoff points of BUN exist in most studies, as evidenced by the fact that eleven patients (1.64%) died during hospitalization in the current study.

Multiparametric scores such as APACHE II score and BISAP score are essential for clinical trials but have obvious disadvantages that prevent their practical use in daily routine. APACHE II score is not specific for pancreatitis and time-consuming to be calculated [6]. BISAP score had a moderate diagnostic accuracy in predicting AP [23]. In addition to the established clinical scores, a variety of single markers such as C-reactive protein [24] or interleukin- (IL-) 6 and IL-8 had predictive value [25] and have emerged for AP. However, interleukin- (IL-) 6 and IL-8 cannot be routinely available in the emergency room. C-reactive protein, though easy to check in practice, lacks high specificity [26].

The strength of this study includes adequate sample size which gives significant statistical power. Only patients with short onset time (not more than three days) of AP and having first attack were enrolled, which might help rule out the influence of interferences such as fluid resuscitation on BUN level. It was the first study to evaluate the prognostic value of BUN via up-to-date revised Atlanta criteria of the SAP. However, there were several potential limitations such as the retrospective design which might produce selection bias. The timing of BUN measurement was only available at admission and 24 hrs after hospital admission, which could not let us evaluate the impact of early changes in BUN on prognosis of AP. PPV could not be close to one for this study, even if both sensitivity and specificity were high [27].

In conclusion, BUN was the most valuable independent risk factor for predicting SAP and IHM in AP during the initial 24 hrs of hospitalization. BUN after 24 hrs of hospital admission showed a better prognostic accuracy than BUN at initial admission for SAP. The optimal cutoff point of BUN at 24 hrs after hospital admission was 8.3 mmol/L (23.2 mg/dL). The best timing for BUN measurement for IHM was at admission, whose optimal cutoff point was 13.3 mmol/L (37.3 mg/dL). BUN as a single marker for AP could be useful as it is easy to perform and a cheap marker to predict SAP and IHM, without the need for complex calculations.

## Figures and Tables

**Table 1:** Univariate analysis of predictive factors of SAP in 671 patients.

Variable	No SAP (*n* = 611)	SAP (*n* = 60)	*P* values
Age (yr)	47 (37, 63)	51.5 (38.5, 66)	0.1441
Male (%)	63.3	53.3	0.1270
Biliary etiology (%)	265 (43.4)	19 (31.7)	
BMI (kg/m^2^)	23.5 (21.1, 26.1)	24.2 (22.1, 26.6)	0.1041
WBC (10^9^/l)	13.2 (10, 16.77)	15.3 (11.09, 18.365)	0.0185
Hemoglobin (g/l)	144 (129, 158)	149.5 (136, 164.5)	0.0722
Platelet (10^9^/l)	198 (233, 160)	172 (209.5, 134)	0.0070
Potassium (mmol/l)	3.98 (3.70, 4.25)	4.02 (3.65, 4.70)	0.1443
Sodium (mmol/l)	139 (141, 136)	137 (142, 133)	0.1390
Calcium (mmol/l)	2.19 (2.28, 2.07)	1.9 (2.195, 1.58)	<0.001
BUN (mmol/l)	4.7 (3.7, 5.9)	7.45 (5.45, 11.95)	<0.001
BUN (24 h) (mmol/l)	4.6 (3.4, 6.2)	9.4 (6.15, 12.35)	<0.001
Scr (umol/L)	64 (54, 76)	82.5 (58, 157.5)	<0.001
Scr (24 h) (umol/L)	63 (52, 77)	92 (66, 206.5)	<0.001

Single continuous variables as median and interquartile range (25th–75th percentiles).

**Table 2:** Index of BUN diagnosis for SAP upon patient admission.

BUN (mmol/L)	BUN (mg/dL)	Youden Index	Sensitivity	Specificity	PPV	NPV
5	14	0.343	0.783	0.560	0.149	0.963
6	17	0.388	0.633	0.755	0.202	0.954
**6.1**	**17.1**	**0.428**	**0.633**	**0.795**	**0.233**	**0.957**
7	20	0.375	0.517	0.858	0.269	0.948
8	22	0.386	0.483	0.903	0.329	0.947
9	25	0.369	0.433	0.936	0.401	0.947
10	28	0.301	0.350	0.951	0.412	0.917
11	31	0.238	0.267	0.971	0.470	0.900
12	34	0.227	0.250	0.977	0.512	0.930

PPV = positive predictive value; NPV = negative predictive value.

**Table 3:** Index of BUN diagnosis for SAP, 24 hrs after hospital admission.

BUN (mmol/L)	BUN (mg/dL)	Youden Index	Sensitivity	Specificity	PPV	NPV
5	14	0.396	0.833	0.563	0.174	0.972
6	17	0.505	0.783	0.722	0.217	0.971
7	20	0.483	0.650	0.833	0.277	0.960
8	22	0.482	0.583	0.899	0.356	0.956
**8.3**	**23.2**	**0.508**	**0.583**	**0.925**	**0.432**	**0.958**
9	25	0.489	0.550	0.939	0.472	0.955
10	28	0.407	0.450	0.957	0.519	0.947
11	31	0.388	0.417	0.971	0.581	0.947
12	34	0.243	0.267	0.976	0.533	0.931

PPV = positive predictive value; NPV = negative predictive value.

**Table 4:** Index of BUN diagnosis for IHM at initial patient admission in the hospital.

BUN (mmol/L)	BUN (mg/dL)	Youden Index	Sensitivity	Specificity	PPV	NPV
5	14	0.538	1	0.538	0.035	1
**5.2**	**14.6**	**0.609**	**1**	**0.609**	**0.041**	**1**
6	17	0.454	0.727	0.727	0.043	0.991
7	20	0.462	0.636	0.826	0.058	0.993
8	22	0.513	0.636	0.877	0.077	0.993
9	25	0.457	0.546	0.911	0.079	0.992
10	28	0.478	0.546	0.932	0.117	0.969
11	31	0.411	0.455	0.956	0.148	0.991
12	34	0.419	0.455	0.964	0.172	0.990
** 13.3**	**37.3**	**0.426**	**0.455**	**0.971**	**0.217**	**0.991**
13.9	38.9	0.337	0.364	0.973	0.190	0.989

PPV = positive predictive value; NPV = negative predictive value.

**Table 5:** Index of BUN diagnosis for IHM, after 24 hrs of patient hospitalization.

BUN (mmol/L)	BUN (mg/dL)	Youden Index	Sensitivity	Specificity	PPV	NPV

8	22	0.592	0.727	0.865	0.082	0.995
**8.3**	**23.2**	**0.616**	**0.727**	**0.889**	**0.099**	**0.995**
9	25	0.541	0.636	0.905	0.104	0.993
10	28	0.566	0.636	0.930	0.132	0.994
11	31	0.582	0.636	0.946	0.163	0.994
** 12**	**34**	**0.508**	**0.546**	**0.962**	**0.205**	**0.989**
13.9	38.9	0.335	0.364	0.971	0.174	0.989
15	42.0	0.247	0.273	0.974	0.150	0.988

PPV = positive predictive value; NPV = negative predictive value.
